# Genetic structures of invasive *Streptococcus pneumoniae* isolates from Korean children obtained between 1995 and 2013

**DOI:** 10.1186/s12879-018-3177-7

**Published:** 2018-06-08

**Authors:** Ki Wook Yun, Eun Hwa Choi, Hoan Jong Lee, Jin Han Kang, Kyung-Hyo Kim, Dong Soo Kim, Yae-Jean Kim, Byung Wook Eun, Sung Hee Oh, Hye-Kyung Cho, Young Jin Hong, Kwang Nam Kim, Nam Hee Kim, Yun-Kyung Kim, Hyunju Lee, Taekjin Lee, Hwang Min Kim, Eun Young Cho, Chun Soo Kim, Su Eun Park, Chi Eun Oh, Dae Sun Jo, Young Youn Choi, Jina Lee

**Affiliations:** 1 Department of Pediatrics, Seoul National University College of Medicine, 101 Daehak-ro, Jongno-gu, Seoul, 03080 South Korea; 2 Department of Pediatrics, College of Medicine, the Catholic University of Korea, Seoul, South Korea; 3 Department of Pediatrics, School of Medicine, Ewha Womans University, Seoul, South Korea; 4 Department of Pediatrics, Yonsei University College of Medicine, Seoul, South Korea; 5 Department of Pediatrics, Sungkyunkwan University School of Medicine, Samsung Medical Center, Seoul, South Korea; 6 Department of Pediatrics, Eulji University School of Medicine, Daejeon, South Korea; 7 Department of Pediatrics, Hanyang University, College of Medicine, Seoul, South Korea; 8 Department of Pediatrics, Graduate School of Medicine, Gachon University, Incheon, South Korea; 9 Department of Pediatrics, Inha University School of Medicine, Incheon, South Korea; 10 Department of Pediatrics, Hallym University College of Medicine, Chuncheon, South Korea; 11 Department of Pediatrics, Inje University College of Medicine, Seoul, South Korea; 12 Department of Pediatrics, Korea University College of Medicine, Seoul, South Korea; 13 Department of Pediatrics, Seoul National University Bundang Hospital, Seoul, South Korea; 14 Department of Pediatrics, CHA Bundang Medical Center, CHA University, Seoul, South Korea; 15 Department of Pediatrics, Yonsei University Wonju College of Medicine, Wonju, South Korea; 16 Department of Pediatrics, Chungnam National University Hospital, Daejeon, South Korea; 17 Department of Pediatrics, Keimyung University School of Medicine, Daegu, South Korea; 18 Department of Pediatrics, Pusan National University School of Medicine, Busan, South Korea; 19 Department of Pediatrics, Kosin University College of Medicine, Busan, South Korea; 20 Department of Pediatrics, Chonbuk National University Medical School, Jeonju, South Korea; 21 Department of Pediatrics, Chonnam National University Medical School, Gwangju, South Korea; 22 Department of Pediatrics, University of Ulsan College of Medicine, Seoul, South Korea

**Keywords:** Invasive pneumococcal diseases, Pneumococcal conjugate vaccine, Multilocus sequence typing, Children

## Abstract

**Background:**

Understanding the population genetics of pneumococci will allow detection of changes in the prevalence of circulating genotypes and evidence for capsular switching. We aimed to analyze the genetic structure of invasive pneumococcal isolates obtained from children before and after the use of pneumococcal conjugate vaccines (PCVs) in Korea.

**Methods:**

A total of 285 invasive pneumococcal isolates were analyzed using serotyping, multilocus sequence typing, and antimicrobial susceptibility testing. We classified the isolation year to pre-PCV7 (1995–2003; *n* = 70), post-PCV7 (2004–2010; *n* = 142), and post-PCV13 (2011–2013; *n* = 73) periods.

**Results:**

Of the 10 clonal complexes (CCs), antibiotic-resistant international clones, CC320 (31.6%), CC81 (14.7%), and CC166 (6.7%) were the main complexes. Serotype 19A was the main serotype of CC320 throughout the periods. Serotypes of CC81 mainly comprised of 23F (53.3%) in pre-PCV7 period and replaced by non-vaccine types (NVTs; 6C [10%], 13 [30%], 15A [40%], and 15B/C [20%]) in post-PCV13 period. The main serotype responsible for CC166 also changed from 9 V (80%) in pre-PCV7 to NVT 11A (50%) in post-PCV13 periods. Non-susceptibility to penicillin (42.3%) was the highest in CC320, increasing from 0 to 76%.

**Conclusion:**

The genetic structures of invasive pneumococcal isolates in Korean children have changed concomitantly with serotype after the implementation of PCVs.

## Background

Pneumococcal conjugate vaccines (PCVs) targeting previously predominant invasive serotypes has been highly successful in decreasing the incidence of invasive pneumococcal disease (IPD) and pneumococcal antimicrobial resistance [1, 2]. However, individual variants that originated through serotype switching have substantially contributed to the disease burden in the post-7-valent PCV (PCV7) era [3]. Additionally, clonal expansion is considered to be a main mechanism for ongoing antibiotic resistance [4]. Since serotype and genotype are closely associated, changes in the circulating serotype have also resulted in a concomitant change in the circulating genotype [5, 6]. Therefore, information on both the genetic background and the capsular serotype allowed us to analyze the responses to vaccine implementation [7, 8].

PCV7 was introduced in Korea in November 2003 for optional use. In June 2010, 10- and 13-valent PCVs (PCV10 and PCV13) were introduced to replace PCV7. In May 2014, both PCV10 and PCV13 were included in the national immunization program (NIP) for children under 60 months of age. Korea is one of a few countries where both PCV10 and PCV13 are included in NIP. Studies on serotype changes in IPD after each PCV7 and PCV10/13 implementation have been conducted [9, 10], but the genetic backgrounds of IPD isolates have been investigated only in the pre-PCV7 era thus far [11]. Therefore, we aimed to characterize the changes in genetic structures of invasive pneumococcal isolates obtained from children before and after the optional use of PCV7 and PCV10/13 in Korea.

## Methods

### Subjects

Hospital-based surveillance to monitor pneumococcal diseases as part of routine clinical care at Seoul National University Children’s Hospital (SNUCH) has been employed since 1991. In 2006, a nation-wide surveillance system to monitor IPD began with 8 teaching hospitals, including SNUCH. In 2011, the surveillance system was expanded to include 25 hospitals throughout Korea. In this study, IPD cases and corresponding pneumococcal isolates obtained from children aged < 18 years were included. This study was approved by the Institutional Review Board of Seoul National University Hospital (IRB registration number 1106–015-364) and each participating institute. Informed consent was waived, because the study was retrospective and the pneumococcal isolates were obtained as a standard part of routine patient care. To explore the changes in genetic background following the implementation of PCV7 and PCV10/13, we grouped the isolation years to pre-PCV7 (1995–2003), post-PCV7 (2004–2010), and post-PCV13 (2011–2013) periods.

### Isolates and serotyping

All pneumococcal isolates obtained from IPD cases were sent to SNUCH, and serotype determination was performed using the Quellung reaction with antisera (Statens Serum Institut, Copenhagen, Denmark) and polymerase chain reaction (PCR) [12]. A case of IPD was defined as an infection confirmed by the isolation of pneumococci from a normally sterile site, such as the blood, cerebrospinal fluid, pleural fluid, ascites, or joint fluid. For multiple isolates collected from a single episode of infection, only the initial isolate was included. Serotypes 4, 6B, 9 V, 14, 18C, 19F, and 23F were classified as PCV7 types. The PCV13 types included six PCV13-additional (aPCV13) serotypes: 1, 3, 5, 6A, 7F, and 19A. The non-PCV types (NVTs) included all other serotypes. All serotype data on study isolates were extracted from previous studies [9–11].

### Multilocus sequence typing

Genomic DNA templates were prepared from pure *Streptococcus pneumoniae* cultures according to the manufacturer’s instructions (Qiagen, Manchester, UK). The sequences of 7 housekeeping genes (*aroe, gdh, gki, recP, spi, xpt*, and *ddl*) were used in the pneumococcal multilocus sequence typing (MLST) scheme as previously described [13]. New alleles and sequence types (STs) were submitted to the MLST web database (https://pubmlst.org) for assignment. eBURST v3 software, which is available on the MLST website, was used to estimate the relationships among the isolates and to assign the strains to a clonal complex (CC) [14]. Single- and double-locus variants (SLVs and DLVs, respectively) were defined as STs differing from the confounding ST at one and two loci, respectively. MLST data on 77 pneumococci isolated from 1995 to 2005 were obtained from a previous IPD study [11]. Data on all serogroup 6 (*n* = 35) were also a part of a previous publication [15].

### Antimicrobial susceptibility testing

A total of 236 pneumococcal isolates were tested to determine the minimal inhibitory concentrations (MICs) of 8 antimicrobial drugs (penicillin, cefotaxime, chloramphenicol, tetracycline, clindamycin, erythromycin, trimethoprim-sulfamethoxazole, and levofloxacin) using the E-test (bioMérieux, Marcy l’Etoile, France). Only MIC data on all isolates (*n* = 73) from 2011 to 2013 were produced in this study, whereas those of isolates from 1995 to 2010 were extracted from previous studies. Forty randomly selected isolates among the 77 invasive pneumococci from 1995 to 2005 [11] and most isolates (*n* = 123) - with the exception of 12 strains that failed to regrow - from 2006 to 2010 [9] were included in the analysis. The breakpoints described in the 2014 Clinical and Laboratory Standards Institute (CLSI) guideline were used [16]. Non-meningitis criteria of parenteral drugs were used for penicillin (2.0 μg/mL) and cefotaxime (1.0 μg/mL).

### Statistical analysis

Statistical analysis was performed using SPSS Statistics, version 23.0 (IBM Corp., Armonk, NY). Rates and proportions were compared using the chi-square test or Fisher’s exact test, where appropriate. A *P* value < 0.05 was considered statistically significant.

## Results

### Isolate and serotype distributions in pre- and post-PCV periods

A total of 285 invasive pneumococcal isolates were included in the analysis: 77 (27.0%) isolates at SNUCH from 1995 to 2005 and 208 (73.0%) isolates at 8 centers from 2006 to 2010 (*n* = 135) and 25 centers from 2011 to 2013 (*n* = 73). Pre-PCV7, post-PCV7, and post-PCV13 periods included 70 (24.6%), 142 (49.8%), and 73 (25.6%) isolates, respectively.

Among the 285 invasive isolates, 280 (98.2%) strains were typeable pneumococci. Of these typeable isolates, a total of 30 serotypes were identified. The most common serotype was 19A (*n* = 64, 22.9%), followed by 23F (*n* = 29, 10.4%), 19F (*n* = 25, 8.9%), 14 (*n* = 23, 8.2%), and 6B (*n* = 23, 8.2%). PCV7 and PCV13 serotypes accounted for 42.1% (*n* = 118) and 73.2% (*n* = 205) of all serotypes, respectively. Throughout the 18-year period, there were significant decreases in the proportions of PCV7 types between the pre-PCV7 (*n* = 45, 67.1%) and post-PCV7 (*n* = 64, 46.7%) periods, and between post-PCV7 and post-PCV13 periods (*n* = 7, 9.6%) (*P* = 0.019 and *P* < 0.001, respectively). The aPCV13 types increased gradually from 21.4% (*n* = 15) in the pre-PCV7 to 32.8% (*n* = 45) in the post-PCV7 period and then to 37.0% (*n* = 27) in the post-PCV13 period (*P* = 0.087 and *P* = 0.547, respectively). NVTs increased significantly, but only between the post-PCV7 (*n* = 28, 20.4%) and post-PCV13 (*n* = 39, 53.4%) periods (*P* < 0.001).

### Genotype diversity of invasive pneumococci recovered from 1995 to 2013

**Fig. 1:**
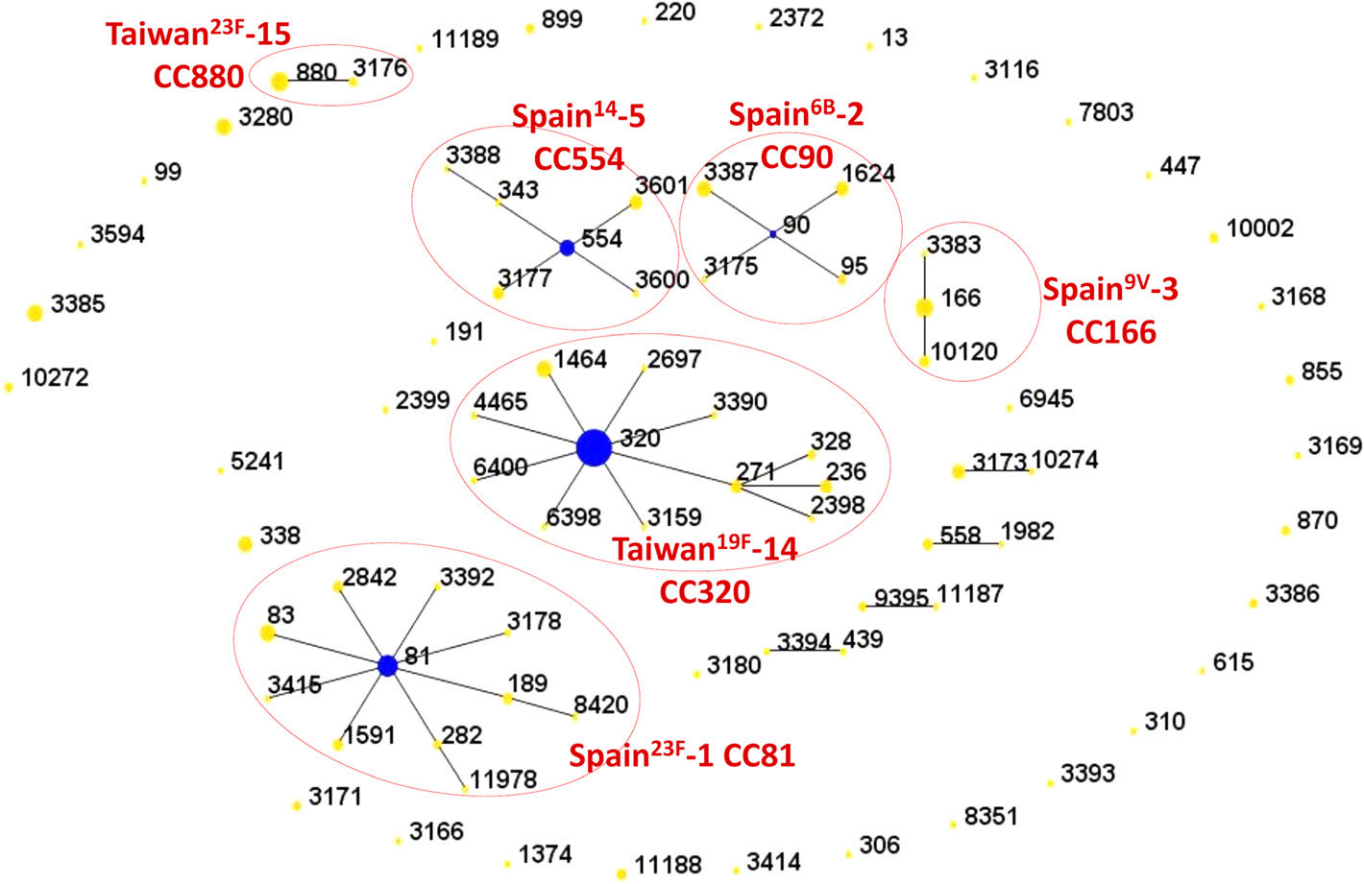
eBURST diagram with the results of multilocus sequence typing of all study isolates (*n* = 285). Circle sizes correlate with the number of isolates of each sequence type. International Pneumococcal Molecular Epidemiology Network clones associated with certain clonal complexes are indicated in red. CC, clonal complex

A total of 81 STs with 10 CCs and 35 singletons were assigned (Fig. 1). CC81, CC90, CC166, CC320, CC554, and CC880 were associated with the well-known international Pneumococcal Molecular Epidemiology Network (PMEN) clones Spain^23F^-1, Spain^6B^-2, Spain^9V^-3, Taiwan^19F^-14, Spain^14^–5, and Taiwan^23F^-15, respectively. Prevalent STs were ST320 (*n* = 66, 23.2%; 19A [*n* = 58], 19F [*n* = 7], 3 [*n* = 1]), ST81 (*n* = 18, 6.3%; 6A [*n* = 8], 23F [*n* = 9], and 14 [*n* = 1]), ST166 (*n* = 15, 5.3%; 9 V [*n* = 9], 11A [*n* = 5], and 15C [*n* = 1]), and ST880 (*n* = 15, 5.3%; all 23F). Among the 10 CCs identified in this study, CC320 (*n* = 90 [31.6%], 11 STs) was the most common, followed by CC81 (*n* = 42 [14.7%], 10 STs) and CC166 (*n* = 19 [6.7%], 3 STs).

### Serotype changes in the main clonal complexes

#### CC320

**Fig. 2:**
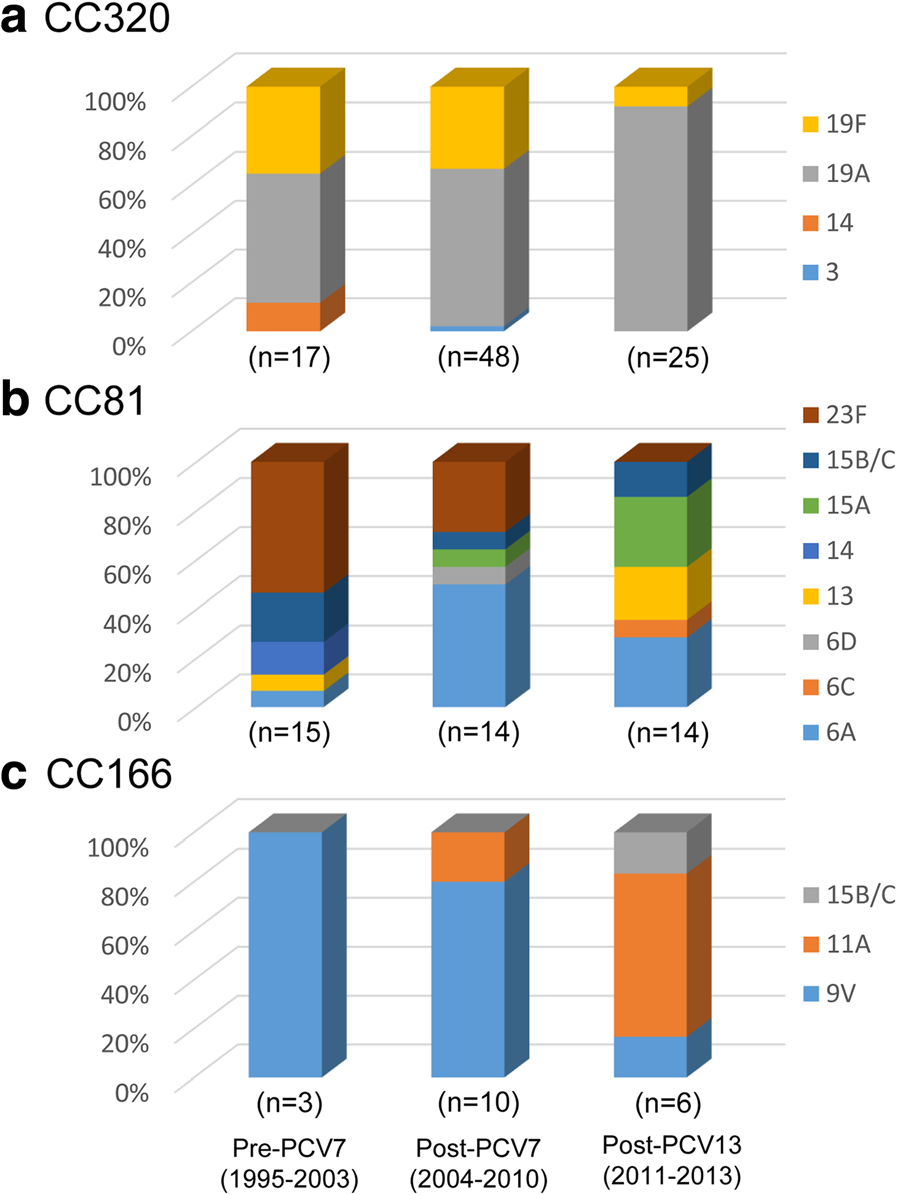
Sequence type changes in the main genotypes CC320 (**a**), CC81 (**b**), and CC166 (**c**) across the study periods. In CC320, serotype 19A increased from 52.9 to 64.6%, and then to 92.0% (**a**). In CC81, serotype 23F decreased significantly, whereas serotypes 6A, 13, and 15A increased (**b**). The proportions of serotypes 9 V and 11A crossed over into CC166 during the study periods (**c**). PCV, pneumococcal conjugate vaccine; MLST, multilocus sequence typing; CC, clonal complex; S, singleton

CC320 remained the most dominant CC throughout all three study periods. CC320 slightly increased from 24.3% (*n* = 17) in the pre-PCV7 to 33.8% (*n* = 48) in the post-PCV7 period (*P* = 0.158) and then remained at 34.2% (*n* = 25) in the post-PCV13 period. The proportion of 19A in CC320 increased from 52.9% (*n* = 9) to 64.6% (*n* = 31) and then to 92.0% (*n* = 23) in the pre-PCV7, post-PCV7, and post-PCV13 periods, respectively (Fig. 2a).

#### CC81

CC81 decreased significantly from 21.4% (*n* = 15) in the pre-PCV7 period to 9.9% (*n* = 14) in the post-PCV7 period (*P* = 0.021) but increased to 19.2% (*n* = 14) in the post-PCV13 period (*P* = 0.055). Serogroups 6, 13, 14, 15, and 23 shared CC81, in which the PCV7 type decreased significantly from 66.7% (*n* = 10; serotypes 14 [*n* = 2] and 23F [*n* = 8]) to 0% (*P* = 0.001), and the aPCV13 type and NVT tended to increase from 6.7% (*n* = 1; serotype 6A) to 36.4% (*n* = 4; all serotype 6A) (*P* = 0.169) and from 28.6% (*n* = 4; serotypes 13 [*n* = 1] and 15B/C [*n* = 3]) to 71.4% (*n* = 10; serotypes 6C [*n* = 1], 13 [*n* = 3], 15A [*n* = 4], and 15B/C [*n* = 2]) (*P* = 0.066), respectively, between the pre-PCV7 and post-PCV13 periods (Fig. 2b).

#### CC166

The prevalence of CC166 almost unchanged between the pre-PCV7 (4.3%, *n* = 3; all 9 V) and post-PCV7 periods (7.0%, *n* = 10; 9 V [*n* = 8] and 11A [*n* = 2]) (*P* = 0.552), and between the post-PCV7 and post-PCV13 periods (8.2%, *n* = 6; 9 V [*n* = 1], 11A [*n* = 3], and 15B/C [*n* = 1]) (*P* = 0.756). The main serotype responsible for CC166 changed from 9 V (100%) to 11A (50%) throughout the study periods (Fig. 2c).

### Genotypic changes in the main serotypes

#### Serogroup 6

In serogroup 6, CC81 and CC90 were the main genotypes (n = 14, 33.3% in both; Table 1). Serotype 6A mainly consisted of CC81 (25.0, 87.5, and 100% in the pre-PCV7, post-PCV7, and post-PCV13 periods, respectively), and those in the singleton STs (ST855, ST3166, and ST3168) disappeared across the study periods. In serotype 6B, the proportion of CC90 increased from 55.6% (*n* = 13) to 100% (n = 1), but the number of total isolates decreased significantly in the post-PCV13 period. One isolate each of serotypes 6C and 6D was first identified in the post-PCV7 period and was assigned to ST5251 and ST11978, respectively, which had not been reported before in serogroup 6, although ST11978 was a ST of CC81. In the post-PCV13 period, one 6C isolate was identified in ST282, which also included the 6A isolate.

**Table 1:** Sequence type changes in individual serotypes during the study periods

Serotype	CC/ST (relation to confounder)	CC percentage (n) of the serotype during			n
		Pre-PCV7 (1995–2003)	Post-PCV7 (2004–2010)	Post-PCV13 (2011–2013)	
1	ST615	0	100 (1)	0	1
	ST306	0	100 (1)	0	1
3	ST10002	0	100 (2)	0	2
	CC320	0	100 (1)	0	1
	ST320	0	100 (1)	0	1
4	ST899	0	100 (2)	0	2
5	CC439	100 (1)	0	0	1
	ST3394 (SLV)	100 (1)	0	0	1
6A	CC81	25.0 (1)	87.5 (7)	100 (4)	12
	ST81	25.0 (1)	62.5 (5)	50.0 (2)	8
	ST282 (SLV)	0	0	25.0 (1)	1
	ST2842 (SLV)	0	25.0 (2)	25.0 (1)	3
	ST855	25.0 (1)	12.5 (1)	0	2
	ST3166	25.0 (1)	0	0	1
	ST3168	25.0 (1)	0	0	1
6B	CC90	55.6 (5)	61.5 (8)	100 (1)	14
	ST90	11.1 (1)	0	0	1
	ST95 (SLV)	22.2 (2)	0	0	2
	ST1624 (SLV)	22.2 (2)	15.4 (2)	100 (1)	5
	ST3175 (SLV)	0	7.7 (1)	0	1
	ST3387 (SLV)	0	38.5 (5)	0	5
	CC3173	11.1 (1)	38.5 (5)	0	6
	ST3173	11.1 (1)	30.8 (4)	0	5
	ST10274 (SLV)	0	7.7 (1)	0	1
	ST3169	11.1 (1)	0	0	1
	ST3171	22.2 (2)	0	0	2
6C	CC81	0	0	100 (1)	1
	ST282 (SLV)	0	0	100 (1)	1
	ST5241	0	100 (1)	0	1
6D	CC81	0	100 (1)	0	1
	ST11978 (DLV)	0	100 (1)	0	1
7F	ST191	0	100 (1)	0	1
9 V	CC166	100 (3)	100 (8)	100 (1)	12
	ST166	66.7 (2)	75.0 (6)	100 (1)	9
	ST3383 (SLV)	33.3 (1)	0	0	1
	ST10120 (SLV)	0	25.0 (2)	0	2
10A	ST3385	0	100 (5)	83.3 (5)	10
	ST11189	0	0	16.7 (1)	1
11A	CC166	0	50.0 (2)	100 (4)	6
	ST166	0	50.0 (2)	75.0 (3)	5
	ST10120 (SLV)	0	0	25.0 (1)	1
	ST99	0	25.0 (1)	0	1
	ST11188	0	25.0 (1)	0	1
12F	ST220	0	100 (1)	0	1
	ST6945	0	0	100 (1)	1
13	CC81	100 (1)	0	100 (3)	4
	ST189 (SLV)	100 (1)	0	66.7 (2)	3
	ST8420 (DLV)	0	0	33.3 (1)	1
14	CC81	18.2 (2)	0	0	2
	ST81	9.1 (1)	0	0	1
	ST3178 (SLV)	9.1 (1)	0	0	1
	CC320	18.2 (2)	0	0	2
	ST328 (DLV)	18.2 (2)	0	0	2
	CC554	54.5 (6)	100 (11)	100 (1)	18
	ST343 (SLV)	9.1 (1)	0	0	1
	ST554	27.3 (3)	18.2 (2)	100 (1)	6
	ST3177 (SLV)	9.1 (1)	27.3 (3)	0	4
	ST3388 (DLV)	9.1 (1)	0	0	1
	ST3600 (SLV)	0	9.1 (1)	0	1
	ST3601 (SLV)	0	45.5 (5)	0	5
	ST13	9.1 (1)	0	0	1
15A	CC81	0	50 (1)	100 (4)	5
	ST83 (SLV)	0	0	50 (2)	2
	ST1591 (SLV)	0	50 (1)	50 (2)	3
	ST3414	0	50 (1)	0	1
15B/C	CC81	100 (3)	20.0 (1)	22.2 (2)	6
	ST83 (SLV)	100 (3)	20.0 (1)	22.2 (2)	6
	CC166	0	0	11.1 (1)	1
	ST166	0	0	11.1 (1)	1
	ST3280	0	80.0 (4)	66.7 (6)	10
16F	ST8351	0	0	100	1
18C	ST870	0	66.7 (2)	0	2
	ST3180	100 (1)	0	0	1
	ST3594	0	33.3 (1)	0	1
19A	CC320	90.0 (9)	100 (31)	100 (23)	63
	ST271 (SLV)	0	0	4.3 (1)	1
	ST320	90.0 (9)	90.3 (28)	91.3 (21)	58
	ST3159 (SLV)	0	0	4.3 (1)	1
	ST4465 (SLV)	0	3.2 (1)	0	1
	ST6398 (SLV)	0	3.2 (1)	0	1
	ST6400 (SLV)	0	3.2 (1)	0	1
	ST1374	10.0 (1)	0	0	1
19F	CC320	85.7 (6)	100 (16)	100 (2)	24
	ST236 (DLV)	42.9 (3)	6.3 (1)	0	4
	ST271 (SLV)	42.9 (3)	0	0	3
	ST320	0	43.8 (7)	0	7
	ST1464 (SLV)	0	43.8 (7)	50.0 (1)	8
	ST2697 (SLV)	0	0	50.0 (1)	1
	ST3390 (SLV)	0	6.3 (1)	0	1
	ST2399	14.3 (1)	0	0	1
23A	ST338	0	100 (4)	50.0 (2)	6
	ST10272	0	0	50.0 (2)	2
23B	CC439	0	0	50.0 (1)	1
	ST439	0	0	50.0 (1)	1
	ST2372	0	0	50.0 (1)	1
23F	CC81	50.0 (8)	36.4 (4)	0	12
	ST81	37.5 (6)	27.3 (3)	0	9
	ST83 (SLV)	0	9.1 (1)	0	1
	ST3392 (SLV)	6.3 (1)	0	0	1
	ST3415 (SLV)	6.3 (1)	0	0	1
	CC880	50.0 (8)	63.6 (7)	100 (2)	17
	ST880	50.0 (8)	54.5 (6)	50.0 (1)	15
	ST3176 (SLV)	0	9.1 (1)	50.0 (1)	2
24F	CC90	50.0 (1)	0	0	1
	ST3387 (SLV)	50.0 (1)	0	0	1
	ST3386	0	0	100 (1)	1
	ST3393	50.0 (1)	0	0	1
33F	ST11188	0	100 (1)	100 (1)	2
34	CC9395	0	100 (3)	0	3
	ST9395	0	66.7 (2)	0	2
	ST11187 (SLV)	0	33.3 (1)	0	1
	ST3116	100 (1)	0	0	1
35B	CC558	100 (1)	100 (1)	0	2
	ST558	100 (1)	100 (1)	0	2
37	ST447	0	0	100 (1)	1
NT	CC90	0	20.0 (1)	0	1
	ST3387 (SLV)	0	20.0 (1)	0	1
	CC558	0	20.0 (1)	0	1
	ST558	0	20.0 (1)	0	1
	ST310	0	20.0 (1)	0	1
	ST447	0	20.0 (1)	0	1
	ST3386	0	20.0 (1)	0	1

*CC* clonal complex, *ST* sequence type, *S* singleton, *PCV* pneumococcal conjugate vaccine, *n* number, *SLV* single-locus variant, *DLV* double-locus variant, *NT* non-typeable

#### Serotype 14

Serotype 14 exhibited a diverse genetic background in the pre-PCV7 period, although CC554 was the main genotype (*n* = 6, 54.5%; Table 1). As in serotype 6B, another PCV7 type, the proportion of CC554 in serotype 14 increased to 100% in both the post-PCV7 (*n* = 11) and post-PCV13 (n = 1) periods, but the total number of isolates dramatically decreased in the post-PCV13 period.

#### Serogroup 15

In serogroup 15, there was only serotype 15B/C in the pre-PCV7 period (n = 3), but serotype 15A appeared thereafter. Most (*n* = 5, 83.3%) of serotype 15A had the CC81 genotype, a main 15B/C genotype in the pre-PCV7 period (Table 1). The 15B/C genotypes became more diverse across the study periods, and ST3280 was the main genotype in the post-PCV7 (*n* = 4, 80.0%) and post-PCV13 (*n* = 6, 66.7%) periods.

#### Serogroup 19

Serogroup 19 was so genetically homogeneous that 97.8% (*n* = 87) of the isolates belonged to only CC320. In particular, ST320 in serotype 19A was significantly prevalent (90.0%, 58/64). The proportion of ST320 in serotype 19A did not change, remaining at 90.0% (*n* = 9, pre-PCV7), 90.3% (*n* = 28, post-PCV7), and 91.3% (*n* = 21, post-PCV13) across the study periods. ST320 appeared in serotype 19F (*n* = 7, 28.0%) in the post-PCV7 period but disappeared in the post-PCV13 period.

#### Serogroup 23

Similar to serotypes 6C/6D and 15A, serotypes 23A/23B appeared in serogroup 23 beginning in the post-PCV7 period (Table 1). Many serotype 23F isolates belonging to CC81 and CC880 were identified in the pre- and post-PCV7 periods but mostly disappeared in the post-PCV13 period. Serotypes 23A and 23B did not share their genetic structures with any other serotypes in this study, including serotype 23F.

### Antimicrobial resistance changes in the main genotypes

Among the genotypes that included more than five isolates, non-susceptibility to penicillin (42.3%) and cefotaxime (62.0%) was the highest in CC320 (serotypes 19A/19F [*n* = 70, 98.6%] and 14 [*n* = 1, 1.4%]; Table 2). All the isolates in CC320 that were non-susceptible to penicillin or cefotaxime belonged to serogroup 19. CC166 (serotypes 9 V [*n* = 10, 58.8%], 11A [*n* = 6, 35.3%], and 15C [*n* = 1, 5.9%]) showed non-susceptibility to penicillin (11.8%) and/or cefotaxime (17.6%). All the isolates non-susceptible to penicillin or cefotaxime in CC166 belonged to serotype 11A.

**Table 2:** Antimicrobial non-susceptibility in the main genotypes

MLST	n	Non-susceptibility (%) to Antimicrobials							
		PCN	CTX	CLP	TCL	CLM	ETM	LFC	TSZ
CC81	37	10.8	0	94.4	97.2	63.9	97.3	0	80.6
CC90	12	0	0	75.0	91.7	100	100	0	100
CC166	17	11.8	17.6	35.3	100	100	100	0	100
CC320	71	42.3	62.0	12.7	100	97.2	100	1.4	87.1
CC554	13	0	0	76.9	84.6	92.3	92.3	0	23.1
CC880	10	0	10	10.0	100	100	100	0	70.0
ST338	6	0	0	0	100	100	100	0	0
ST3280	9	0	0	0	100	0	100	0	100
ST3385	8	0	12.5	12.5	12.5	0	12.5	0	0

*MLST* multilocus sequence typing, *CC* clonal complex, *ST* sequence type, *n* number, *PCN* penicillin, *CTX* cefotaxime, *CLP* chloramphenicol, *TCL* tetracycline, *CLM* clindamycin, *ETM* erythromycin, *LFC* levofloxacin, *TSZ* trimethoprim-sulfamethoxazole

In contrast, non-susceptibility to chloramphenicol was the highest in CC81 (94.4%; serotypes 6A/6C/6D [*n* = 13, 36.1%], 13 [*n* = 4, 11.1%], 14 [*n* = 1, 2.8%], 15A/15C [*n* = 8, 22.2%], and 23F [*n* = 10, 27.8%]) and the lowest in CC880 (10.0%; all 23F [*n* = 10, 100%]). Serotype 23F isolates in CC81 were all resistant to chloramphenicol, but all except one isolate in CC880 were susceptible to the antibiotic. Most isolates were highly non-susceptible to tetracycline, clindamycin, and erythromycin, whereas most study isolates except for one in CC320 (an isolate in serotype 19A/ST320) were exclusively susceptible to levofloxacin. CC166 and ST3280 (all serotype 15B/C) were non-susceptible to trimethoprim-sulfamethoxazole. Overall, CC320 showed the most multi-drug resistance, whereas ST3385 (all serotype 10A) showed the most susceptible patterns.

In CC320, all isolates were susceptible to penicillin and cefotaxime in the pre-PCV7 period; however, non-susceptibility increased to 76.0 and 68.0%, respectively, in the post-PCV13 period. After applying the alternative breakpoints, all isolates were non-susceptible to oral penicillin (0.06 μg/mL) throughout the three study periods, and a similar trend was observed in cefotaxime susceptibility with meningitis breakpoint (0.5 μg/mL). Among the isolates with MIC data in CC320, the proportions of serotype 19A were 0, 67.4% (*n* = 29), and 92.0% (*n* = 23) in the pre-PCV7, post-PCV7, and post-PCV13 periods, respectively. However, serotypes 19A (67.4%) and 19F (32.6%) in CC320 did not exhibit significant differences in non-susceptibility to penicillin (27.6 and 21.4%, respectively; *P* = 1.000) or cefotaxime (58.6 and 71.4%, respectively; *P* = 0.512) in post-PCV7 period. Serotype 19A/CC320 exhibited a significant increase in non-susceptibility to penicillin (27.6 and 73.9%, respectively; *P* = 0.001) in the post-PCV7 and post-PCV13 periods, although the increase in non-susceptibility to cefotaxime was not significant (58.6 and 69.6%, respectively; *P* = 0.416).

## Discussion

In this study, we investigated the genetic structures of 285 strains of *S. pneumoniae* isolated from Korean children with IPD between 1995 and 2013. CC320, the most common and antibiotic-resistant genotype, increased in prevalence since the pre-PCV7 era, but the increase nearly plateaued in the post-PCV13 era. However, non-susceptibility to penicillin and cefotaxime increased across the study periods among the strains within CC320. After implementing PCV7 and PCV10/13, the genotypes of PCV7 type invasive pneumococci isolated from children during the pre-PCV7 period mostly disappeared (CC90, CC554, and CC880) and were re-occupied by PCV13 types (CC320) or NVTs (CC81 and CC166) in the post-PCV13 period.

The genetic structures of clinical pneumococcal isolates in Korea were previously investigated in children under 5 years of age between 1995 and 2005 [11]. The study included 77 invasive and 201 non-invasive pneumococcal isolates, and the most prevalent clone was ST320 (14.4%), which consisted of serotypes 19A (87.5%) and 19F (12.5%). Other common clones were all associated with PCV7 serotypes: ST81 (9.7%, 23F [81.5%]), ST880 (9.4%, 23F [100%]), ST271 (5.0%, 19F [100%]), ST166 (4.3%, 9 V [75.0%]), ST236 (4.3%, 19F [100%]), and ST1464 (4.0%, 19F [100%]). Also among the invasive isolates examined in the current study, CC320 (including ST320, ST271, and ST236), CC81, and CC166 were the most common genotypes in the pre-PCV7 period (1995–2003), and they were largely occupied with the PCV7 types 14/19F (*n* = 8, 47.1%), 23F (n = 8, 53.3%), and 9 V (*n* = 3, 100%), respectively.

The prevalence of the main CCs (CC320 and CC81) did not change significantly in the post-PCV13 period (34.2 and 17.8%, respectively) compared to the pre-PCV7 period (24.3 and 21.4%, respectively). However, the main serotypes in the pre-PCV7 period within CC320 and CC81 (19F and 23F, respectively) were replaced by serotype 19A and serogroups 6 and 15, respectively. In CC320, serotype 19A (*n* = 9, 52.9%; aPCV13 type) was the main serotype in the pre-PCV7 period; its proportion increased to 66.0% in the post-PCV7 and to 92.0% in the post-PCV13 period. CC90, CC554, and CC880 also decreased due to the decreases in serotypes 6B, 14, and 23F, respectively, which all belonged to the PCV7 serotype. CC166, the main CC of serotype 9 V in the pre-PCV7 period, increased because it was re-occupied by serotypes 11A and 15C. In the other studies [17, 18], PCV13 decreased the frequency of PCV13 serotype-associated clones, and clonal shift was particularly detected in the serotype 19A population. During the years 2007–2012 in Canada, which implemented PCV13 in November 2010, CC320 and CC199 decreased to almost 0% of the total isolates by 2012, whereas CC695 and CC191 became the predominant genotypes among IPD in the same year. Several newly appeared STs found in serotypes 19A and 15B/C indicating the possibility of recent serotype switching events [18].

Serotype 19A has replaced many serotypes after the introduction of PCV7 and is shown to be resistant to penicillin; this change is believed to be associated mostly with the clonal expansion of ST320 and ST199 [19, 20]. ST320 emerged as a predominant 19A clone after the introduction of PCV7 but was nearly eliminated after the change to PCV13 in the US. Therefore, most 19A isolates were assigned to ST199 and ST695 in the early PCV13 era [17, 21]. In clinical isolates collected from children in Canada with IPD during 2007–2012, a decline in 19A was observed in 2012. Additionally, a clonal shift was detected in the 19A population as CC320 and CC199 declined, whereas CC695 rose to a majority in 2012, one year after the implementation of PCV13 [18]. However, in Korea, 19A/ST320 was already increased before PCV7 use [22] and has remained a prevalent (*n* = 21, 28.8%) clone in the post-PCV13 era, which may be explained by the study period shortly after the implementation of PCV13 in this study. However, serotype 19A in IPD decreased from 2011 to 2013 [10], so that 19A/ST320 was expected to decrease rapidly thereafter, especially after the use of PCV13 in the NIP.

The pattern of genotypic changes in serogroup 6 was similar to that in serotype 14, as the numbers of isolates and STs both decreased. CC90, the main genotype of serotype 6B, mostly disappeared, whereas CC81, which was previously associated with serotype 6A, was increased in the post-PCV13 period. Although PCV7 (containing 6B) and PCV13 (containing 6A/6B) were considered to be cross-protective to serotypes 6A and 6C, respectively [23, 24], the effect was not prominent in this study. Serotypes 6C in ST282 (post-PCV13 period) and 6D in ST11978 (post-PCV7 period) may have developed on the genetic basis of serotype 6A in CC81, as previously suggested [15].

In contrast to serogroups 6 and 14, serogroup 15 was increased in the numbers of isolates, STs, and serotypes across the study periods. Serotype 15B/C was expanded to three STs (ST83, ST166, and ST3171). Serotype 15A appeared in the post-PCV7 period and then expanded in the post-PCV13 period. Serotypes 15A and 15B/C occupied half of CC81, the genotype of the highly successful MDR PMEN-1 clone. Serotype 15B/C showed increased antimicrobial resistance and genetic complexity worldwide and emerged in the post-PCV13 era [17, 18, 25].

Serotype 23F decreased stepwise after the use of PCV7 and PCV13, and serotypes 23A and 23B appeared in unrelated STs (ST338 and ST439/ST2372, respectively) with other serotypes, including 23F. However, ST338 and ST439 both consisted of serotype 23F in the 1900s and became the main genotype of serotypes 23A and 23B, respectively, according to the MLST database. Since 2008, ST2372 has only been reported in serotype 23B in Europe. Therefore, serotypes 23A/23B may be imported instead of capsular switching with isolates of other serotypes in Korea. Similar to serogroup 15, serotype 23A has been noted recently in invasive diseases [26].

As mentioned before, in the US, PCV13 effectively decreased antimicrobial resistance, primarily due to the elimination of the resistant serotype 19A/ST320. The strain that contributes significantly to β-lactam resistance since 2011–2013 is now 35B/ST558. Thus, clonal expansion of the newly introduced resistant clone is considered to be the main mechanism for the increase in antibiotic resistance after the introduction of PCV13 in the US [4, 17]. However, in our study, there was a significant increase in the non-susceptibility rate to penicillin within a single clone, 19A/ST320 in post-PCV13 period compared to pre-PCV13 period, suggesting other factors such as high exposure to antimicrobials contribute to the increase in MICs against penicillin within a single drug-resistant clone.

## Conclusions

This study was limited by including uneven data from three different surveillance systems in the study period. However, this was the largest and longest study on the molecular epidemiology of IPD in Korean children. We showed a comprehensive view of the genetic structures of IPD isolates among children spanning the optional use of PCV7 and PCV10/13 in Korea. Ongoing surveillance and research on changes in the molecular epidemiology of IPD after the introduction of PCV10/13 into the NIP in 2014 is expected to elucidate the mechanisms of serotype and antimicrobial changes in more detail and to reveal emerging serotypes in IPD in children.

## Abbreviations

aPCV13: PCV13-additional type
CC: Clonal complex
CLSI: Clinical and Laboratory Standards Institute
DLV: Double-locus variant
IPD: Invasive pneumococcal disease
MIC: Minimal inhibitory concentration
MLST: Multilocus sequence typing
NIP: National immunization program
NVT: Non-vaccine type
PCR: Polymerase chain reaction
PCV: Pneumococcal conjugate vaccine
PCV10: 10-valent pneumococcal conjugate vaccine
PCV13: 13-valent pneumococcal conjugate vaccine
PCV7: 7-valent pneumococcal conjugate vaccine
PMEN: Pneumococcal Molecular Epidemiology Network
SLV: Single-locus variant
SNUCH: Seoul National University Children’s Hospital
ST: Sequence type
